# Development of a Segmental Bioelectrical Impedance Spectroscopy Device for Body Composition Measurement

**DOI:** 10.3390/s19224825

**Published:** 2019-11-06

**Authors:** Thomas Cannon, JungHun Choi

**Affiliations:** Department of Mechanical Engineering, Georgia Southern University, Statesboro, GA 30458, USA; tc05196@georgiasouthern.edu

**Keywords:** bioelectrical impedance spectroscopy, bioimpedance, segmental, BIS, BIA, body composition

## Abstract

Whole-body bioelectrical impedance analysis for measuring body composition has been well-explored but may not be sensitive enough to changes in the trunk compared to changes in the limbs. Measuring individual body segments can address this issue. A segmental bioelectrical impedance spectroscopy device (SBISD) was designed for body composition measurement and a prototype was implemented. Compensation was performed to adjust the measured values to correct for a phase difference at high frequencies and to counteract the hook effect when measuring the human body. The SBISD was used to measure five subjects and was compared against three existing analyzers. For most segmental measurements, the SBISD was within 10% of the R_0_ and R_∞_ values determined with a Bodystat Multiscan 5000 and an Impedimed SFB7. The impedance values from the third reference device, a Seca 514, differed significantly due to its eight-electrode measuring technique, meaning impedance measurements could not be compared directly.

ClinicalTrials.gov Identifier: NCT04006899. Retrospectively registered 1 July 2019 at https://clinicaltrials.gov/ct2/show/NCT04006899

## 1. Introduction

Measuring and tracking body composition can help to assess and track the health of an individual in areas such as nutrition, hydration, and recovery monitoring [1]. Bioelectrical impedance analysis (BIA) has been explored to address these topics. This technique measures the impedance of the body, typically from wrist to ankle, when subjected to a small current injected into the body. Typically, this current is applied with gel tab electrodes or hand grips and foot pads. Several technologies for applying bioelectrical impedance analysis have been developed, differing in the number of frequencies the impedance is measured at, the number of electrodes used, and the sections of the body that are measured. Bioelectrical impedance spectroscopy (BIS) involves taking impedance measurements at many frequencies. BIS, particularly when analyzing individual body segments, is a BIA technique that has not been fully explored, and could allow for a noninvasive, quick, portable, and relatively inexpensive method for determining and tracking body composition. The resistance and reactance at each frequency are plotted and then fitted with a curve, called a Cole–Cole curve, to allow for extrapolation of the body resistance at a theoretical zero frequency, R_0_, and the body resistance at a theoretical infinite frequency, R_∞_ [1].

### 1.1. Pros and Cons of Bioelectrical Impedance Analysis Methods

Compared to many reference methods, bioelectrical impedance analysis methods, such as BIS, are noninvasive, safe, portable, inexpensive, and repeatable, making them ideal for tracking changes [2,3]. It just requires the placement of electrodes on the subject. It has advantages over other techniques, such as dilution methods, dual-energy X-ray absorptiometry (DXA), and air displacement plethysmography (ADP). Dilution techniques require blood samples. Methods like DXA require the baby to be exposed to radiation. BIS is relatively quick and convenient compared with ADP, another measurement option [3]. BIS was found to be capable of measuring the body composition of infants without turning off monitoring equipment in the intensive care unit (ICU) with good repeatability [4]. In that study, the data points at 52.9 kHz, near the operating frequency of a cardio-respiratory monitor, and 26.4 kHz were slight outliers, but with the many frequencies measured, it did not affect results. Single frequency devices would not be capable for such an application due to their focus on the 50 kHz frequency. A survey of BIA studies found that it provides excellent reproducibility for body fat percentage (%BF) estimations, as well as correlation with fat mass (FM) and fat-free mass (FFM) estimations, when compared with reference methods [5]. The surveyors note that the reproducibility is due to easy handling of the devices and there was little variation due to the handler. The majority of the studies in the survey were single-frequency studies; however, 5 of the 30 studies used multi-frequency BIA (MF-BIA) and BIS. Whole body BIA is not sensitive to fluid changes in the trunk, which segmental BIA addresses [6]. Additionally, whole body BIA assumes the individual regions of the body do not significantly differ in composition, which is not necessarily the case [7]. The relative limb lengths of some subjects may differ from the population used for population equations, which whole-body BIA may not address [8]. One study found that, on an individual basis, whole-body BIS did not significantly improve on anthropometry alone [9]. The authors of that study only noted slight improvements and believe that some added information about compartments or segments, such as lengths or diameters, may improve predictions. Another study compared whole-body and segmental BIS to evaluate the body composition of subject with a pitting edema in one leg, before and after a surgery [10]. In that study, whole-body BIA was performed twice, once for the right side and once for the left side of the body, and the authors found that whole-body BIA was appropriate for overall body composition only when measuring on the side opposite the affected leg. Whole-body BIA tends to underestimate fat mass in the trunk, and a potential reason based on trunk circumference was explored [11]. The authors note that in those who are overweight, soft tissue hydration is significantly different. In addition, variations in body shape from the population the prediction equation is based on will affect the result.

### 1.2. Segmental BIA Background

Segmental bioelectric impedance analysis provides an alternative to the BIA methods that analyze the body as a whole. Instead of modelling the whole body as a single cylinder in prediction equations, individual segments are modeled independently, such as the arms, trunk, and legs; however, a variety of other methods using more segments or focusing on certain segments have also been explored [6,8]. There are a variety of methods for placing electrodes for segmental measurements. The current electrodes may be moved to the segment, though it was noted that the sum of the segment measurements was greater than the corresponding whole-body measurement [12]. Segmental measurements may be achieved by placing electrodes at both sides, at each hand and foot, instead of just on one side, as is the case in whole-body measurements [7]. Another option is placing voltage sensing electrodes at the shoulder and upper anterior iliac spine, in addition to the wrist and ankle electrodes [7,13]. Some scales are available that measure foot-to-foot impedance while measuring body weight, providing body composition measurements [12]. Additional devices that provide measurements while the subject is standing have hand grips and foot pads that allow for segmental measurements [14]. These devices provide significantly larger impedance measurements to the more traditional method with wrist and ankle electrodes. This is because these methods include measurement of the hands, wrists, feet, and ankles, while the traditional method starts after the boniest parts of the wrist and ankle. The wrist and ankle combined can have resistances over half that of the whole-body resistance, despite contributing only around 1.8% to the body weight [15].

### 1.3. Purpose

Segmental BIS appears to be a suitable method for keeping the positives of BIA, while addressing the issues with sensitivity to changes in the trunk found in whole-body analysis. The purpose of this preliminary study was to validate the design of a new segmental BIS device (SBISD) capable of measuring the whole body, as well as the individual arm, trunk, and leg segments. The impedance measurements from the SBISD were directly compared against three existing whole-body BIS devices, including the Impedimed SFB7, the gold standard for whole-body BIS devices. The body resistances R_0_ and R_∞_ determined with the SBISD should be within 10% of a reference bioelectrical impedance analyzer.

## 2. Material and Methods

### 2.1. Materials

**Figure 1:**
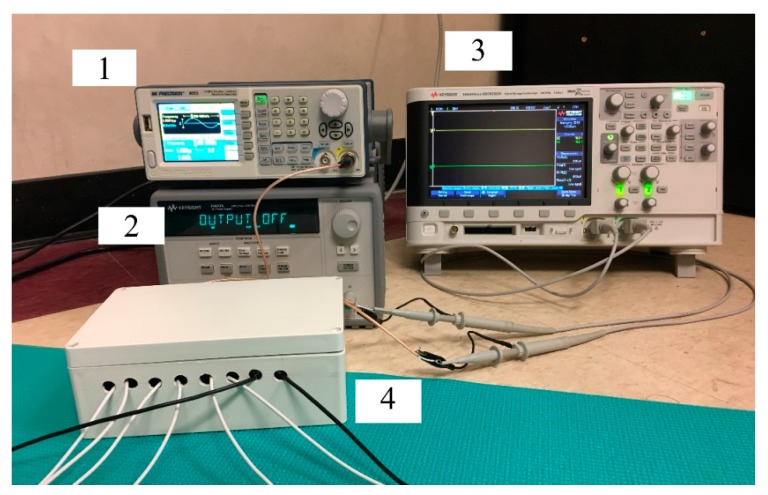
Test equipment and the segmental bioelectrical impedance spectroscopy device (SBISD): (1) signal generator, (2) power supply, (3) digital oscilloscope, and (4) SBISD.

The test equipment and prototype device, referred to as the SBISD from now on, are presented in Figure 1. A signal generator (1) was used to supply a 1 V_pp_ sine wave. A benchtop power supply (2) was used to power the op-amps. A digital oscilloscope (3) was used to measure the peak-to-peak voltage of two signals and the phase difference of the SBISD (4) outputs.

Body composition measurements were performed with a Bodystat Multiscan 5000 (Bodystat Ltd., Douglas, Isle of Man), the Seca 514 Medical Body Composition Analyzer (seca GmbH & Co. KG., Hamburg, Germany), and SFB7 (Impedimed Ltd., Pinkenba, QLD, Australia).

### 2.2. SBISD Prototype for Whole-Body Measurement

**Figure 2:**
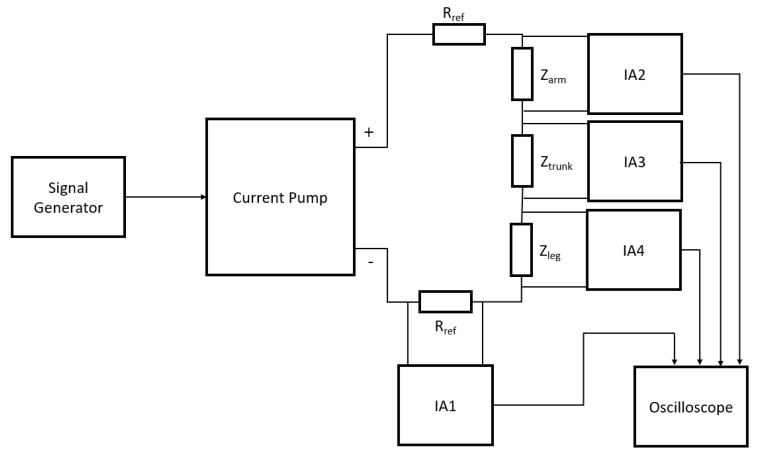
Block diagram for segmental measurements. IA—instrumentation amplifier.

An initial prototype of the SBISD was constructed for whole-body measurement so that it could be validated. For prototype testing, a signal generator was used to provide a 1 V_pp_ sine wave from 3 kHz to 1 MHz. This signal was input to the current pump, which was designed to provide a constant current of 280 μA_pp_ given a 1 V_pp_ input. The ends of the current pump were attached to reference resistors, R_ref_, which were selected to be 470 Ω so that an appropriately large voltage signal could be measured. Then, leads were attached to the other ends of the reference resistors. These leads connected to the object under test, Z_test_, for injecting current. Multiple instrumentation amplifier (IA) circuits were constructed so that the signal would be amplified to twice the magnitude of the original. The first, IA1, measured the voltage across R_ref_ for a reference measurement. The second, IA2, had leads attached and measured the voltage across Z_test_. The outputs of the IAs was connected to the oscilloscope, which was used to measure the peak-to-peak voltage of each output and the phase difference between the two signals. Two more instrumentation amplifiers were added so that segmental measurements could be performed. The block diagram is presented in Figure 2. With these additions, Z_test_ was separated into three separate segments, allowing for individual measurement of the arm, trunk, and leg in humans.

### 2.3. Validation with Existing Devices

After completion of the prototype SBISD, a trial in humans was approved by the Georgia Southern University Institutional Review Board. Subjects volunteered and then signed a consent form to participate and allow for the sharing of data. The five subjects had a mean body mass of 68.8 kg with a standard deviation of 7.9 kg. The mean height was 178 cm with a standard deviation of 5.3 cm. The mean age of the subjects was 24 years, with a standard deviation of 4 years.

Along with the SBISD, several existing BIA devices were used for validating its impedance measurements. The first device used was the Seca 514. The next device used was the Bodystat Multiscan 5000. The third commercial device used was the Impedimed SFB7. It also required the subject lay supine. Finally, the SBISD was used. Measurements were first performed with the Seca 514. The Seca 514 has a built-in scale, with which subject body mass was recorded. This scale has an accuracy of ±0.3% in the range of 35 kg to 300 kg [16]. Subject height was entered and then a BIA measurement was taken. The raw impedance data provided by the device was recorded. Next, the Multiscan 5000 was used to measure whole-body impedance. Gel tab electrodes were placed at the hand and foot following the guidelines from Bodystat, and the same height and body mass were entered as were used for the Seca 514 measurement. The subject lay supine for 5 min and then the measurement was performed. Then, the SFB7 was used to perform the same measurements, with the same electrodes as the Multiscan 5000 and the same height and body mass as used in the Seca 514 measurement. Finally, the SBISD was used to measure whole-body impedance with the same electrodes as the Multiscan 5000 and SFB7. The average impedance for each value was plotted in MATLAB (MathWorks, Natick, MA, USA), then a circle fit was performed. The resistances where this circle crossed the *x*-axis, R_0_, and R_∞_, were recorded.

### 2.4. Segmental Validation

**Figure 3:**
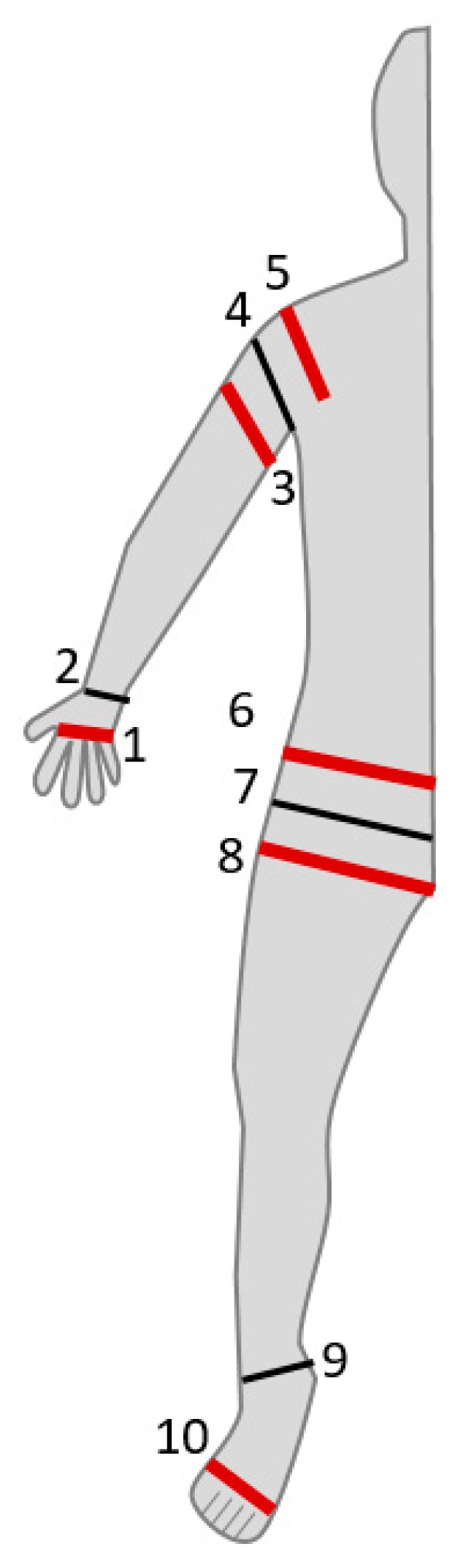
Electrode placements for segmental measurements.

The SBISD was compared with the Seca 514, Multiscan 5000, and SFB7 for segmental measurements. The Seca 514 reports segment data for each arm and leg, as well as the right and left side of the body and the torso. Since all of these were reported at the same time, the segmental data from the whole-body procedure was used. Additional electrodes were placed for measuring individual body segments, including the arm, trunk, and leg. These electrode placements are presented in Figure 3. Voltage sensing electrodes were added at the shoulder and waist, at locations 4 and 7, respectively. The remaining electrodes added—electrodes 3, 5, 6, and 8—were used for current injection, as the leads of the Multiscan 5000 did not allow for being left at the hand and foot for all measurements. Segmental measurements were recorded three times for each segment with the Multiscan 5000.

## 3. Results 

### Measuring Subjects 1 to 5

**Figure 4:**
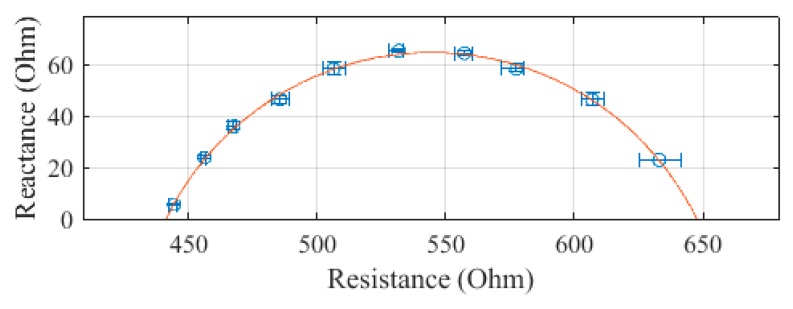
Cole–Cole plot of the whole-body impedance of subject 1 measured with the SBISD.

**Figure 5:**
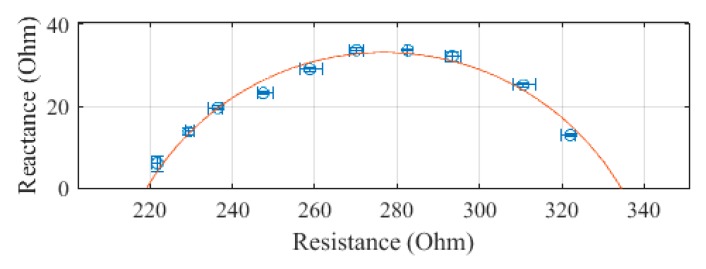
Cole–Cole plot of the arm impedance of subject 1 measured with the SBISD.

**Figure 6:**
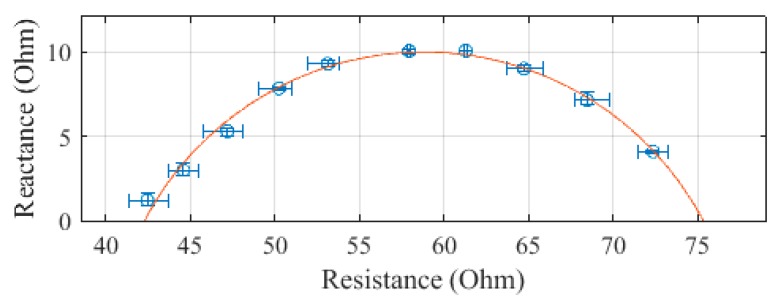
Cole–Cole plot of the trunk impedance of subject 1 measured with the SBISD.

**Figure 7:**
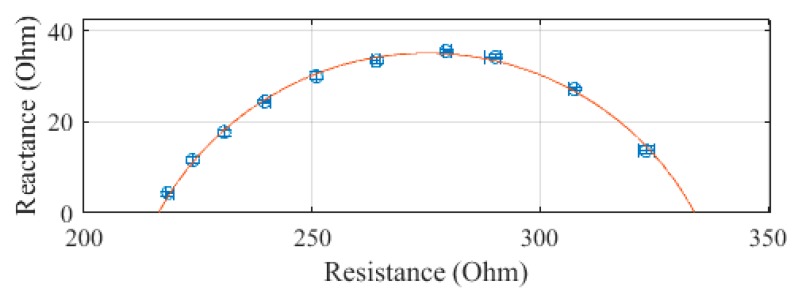
Cole–Cole plot of the leg impedance of subject 1 measured with the SBISD.

The SBISD was used to measure the arm, leg, trunk, and whole body. The first set of leads was attached to the arm, the second to the trunk, and the third to the leg. Then, the second set of leads was used to measure the whole body. Each measurement was repeated for a total of three measurements. The average resistance and reactance values of subject 1 are presented in Figure 4, Figure 5, Figure 6 and Figure 7, with error bars showing the spread of the measurements. The whole body, presented in Figure 4, was determined to have an R_0_ of 647.6 Ω and an R_∞_ of 441.2 Ω. The standard error of the estimate (SEE) for the whole body was calculated to be 1.0 Ω.

The arm, plotted in Figure 5, was measured to have an R_0_ of 334.4 Ω and an R_∞_ of 219.2 Ω. The SEE was calculated to be 1.6 Ω for the arm segment.

The trunk, plotted in Figure 6, was measured to have an R_0_ of 75.3 Ω and an R_∞_ of 42.3 Ω. The SEE was calculated to be 0.4 Ω for the trunk segment.

The leg, plotted in Figure 7, was measured to have a R_0_ of 333.9 Ω and an R_∞_ of 216.5 Ω. The SEE was calculated to be 0.7 Ω for the leg segment.

**Figure 8:**
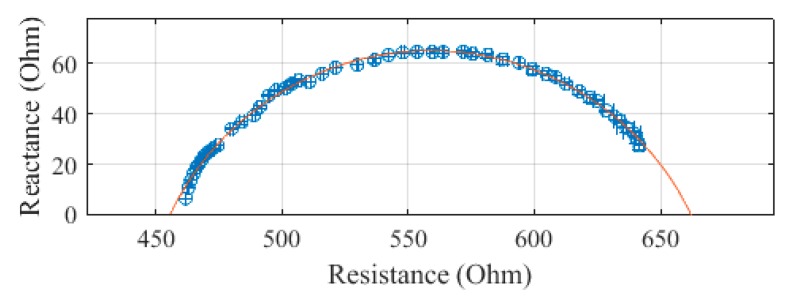
Cole–Cole plot of the whole-body impedance of subject 1 measured with a Multiscan 5000.

**Figure 9:**
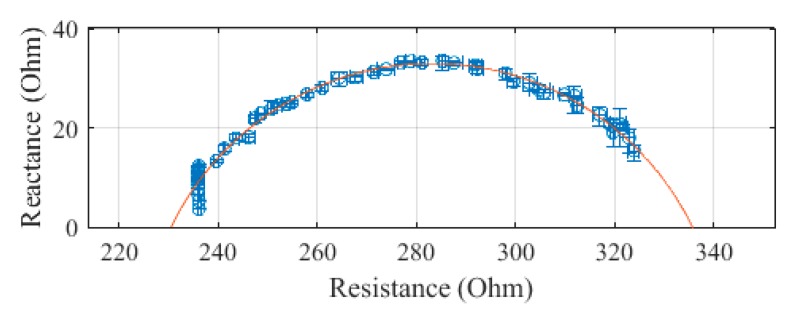
Cole–Cole plot of the arm impedance of subject 1 measured with a Multiscan 5000.

**Figure 10:**
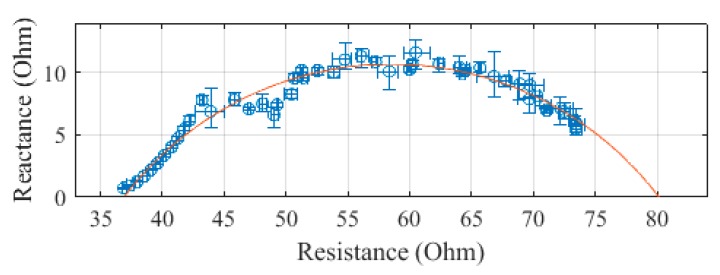
Cole–Cole plot of the trunk impedance of subject 1 measured with a Multiscan 5000.

**Figure 11:**
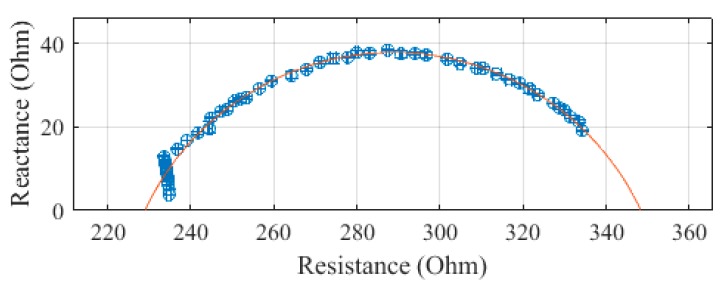
Cole–Cole plot of the leg impedance of subject 1 measured with a Multiscan 5000.

The same segments were measured with the Multiscan 5000, with each measurement repeated three times. The Cole–Cole plots of these measurements on subject 1 are presented in Figure 8, Figure 9, Figure 10 and Figure 11. The average of the three measurements was plotted, with error bars to show the spread of the measurements. The whole body, plotted in Figure 8, was determined to have an R_0_ of 662.0 Ω and an R_∞_ of 455.8 Ω. There was an SEE of 1.2 Ω for the whole body.

The arm was measured to have an R_0_ of 335.7 Ω and an R_∞_ of 230.5 Ω. The high frequencies deviated from the fit circle, leading to an SEE of 1.5 Ω.

The trunk was measured to have an R_0_ of 80.1 Ω and an R_∞_ of 36.9 Ω. The SEE for the trunk segment was calculated to be 0.7 Ω.

The leg was measured to have an R_0_ of 348.3 Ω and an R_∞_ of 229.0 Ω. The leg segment was calculated to have an SEE of 1.7 Ω.

**Figure 12:**
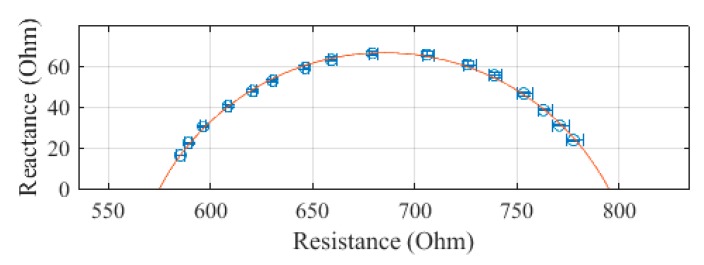
Cole–Cole plot of the whole-body impedance of subject 1 measured with a Seca 514.

**Figure 13:**
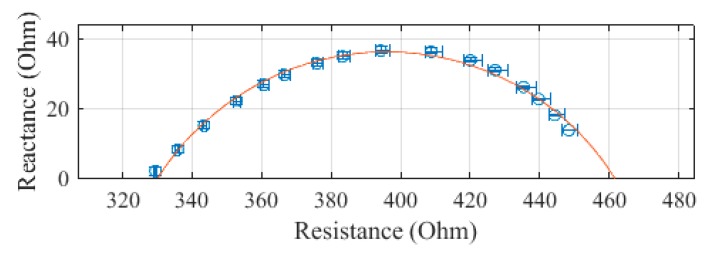
Cole–Cole plot of the arm impedance of subject 1 measured with a Seca 514.

**Figure 14:**
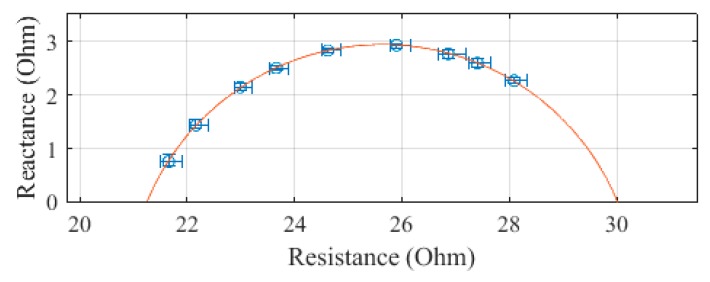
Cole–Cole plot of the trunk impedance of subject 1 measured with a Seca 514.

**Figure 15:**
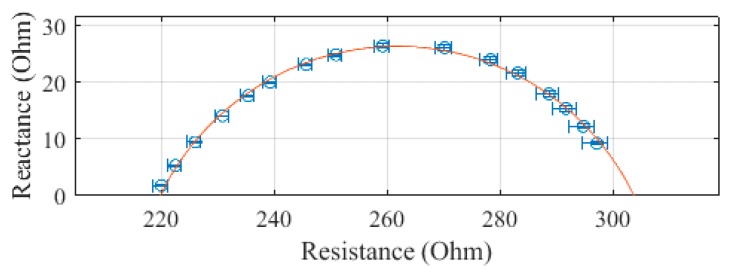
Cole–Cole plot of the leg impedance of subject 1 measured with a Seca 514.

The second device used at this stage was the Seca 514. This device provides the resistance and reactance values for the whole body and each segment after each measurement. The arm and leg segments were compensated to account for the hook effect in Excel 2016 (Microsoft Corp., Redmond, WA, USA), and the measurements below 3 kHz were discarded. The Cole–Cole plots of the average measurement for each segment of subject 1 are presented in Figure 12, Figure 13, Figure 14 and Figure 15. The whole body was determined to have an R_0_ of 795.0 Ω and an R_∞_ of 575.0 Ω. The SEE of the whole-body measurement was calculated to be 0.9 Ω.

The arm was measured to have an R_0_ of 461.6 Ω and an R_∞_ of 330.2 Ω. The SEE was calculated to be 1.0 Ω for the arm segment.

For the torso, measurements above 300 kHz were discarded due to large errors. The trunk was measured to have an R_0_ of 30.0 Ω and an R_∞_ of 21.3 Ω. The SEE, after removing the points, was calculated to be 0.01 Ω.

The leg was measured to have an R_0_ of 303.7 Ω and an R_∞_ of 219.9 Ω. The SEE for the leg segment was calculated to be 0.6 Ω.

**Figure 16:**
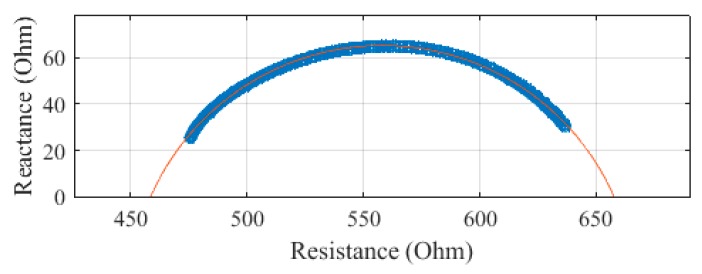
Cole–Cole plot of the whole-body impedance of subject 1 measured with a SFB7.

**Figure 17:**
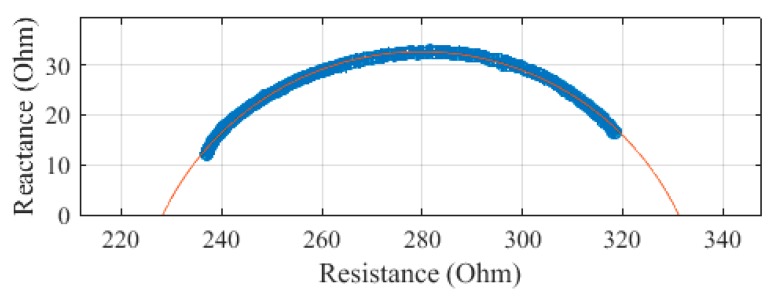
Cole–Cole plot of the arm impedance of subject 1 measured with a SFB7.

**Figure 18:**
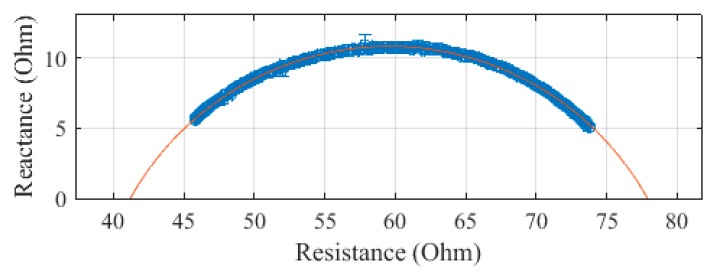
Cole–Cole plot of the trunk impedance of subject 1 measured with a SFB7.

**Figure 19:**
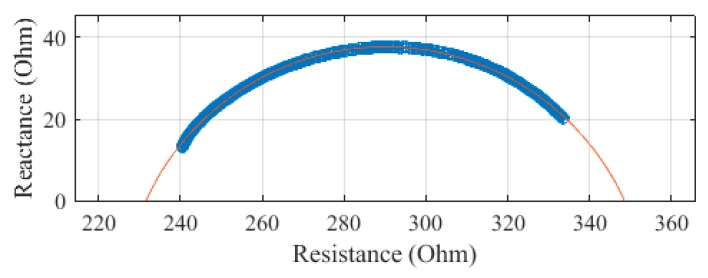
Cole–Cole plot of the leg impedance of subject 1 measured with a SFB7.

Finally, the whole body and each segment were also measured with the SFB7. The Cole–Cole plots for each segment of subject 1 are presented in Figure 16, Figure 17, Figure 18 and Figure 19. Each data point is the average impedance measured at a frequency, with the error bars indicating the spread of the data. The SFB7 software marked data points to be discarded, so these data points were discarded before plotting, reducing the data to the range of 5 kHz to 500 kHz. In this frequency range, R_0_ was found to be 657.7 Ω, R_∞_ was found to be 458.6 Ω, and the SEE was calculated to be 0.6 Ω for the whole-body measurement.

The arm was measured to have an R_0_ of 331.2 Ω and an R_∞_ of 228.3 Ω. The SEE for the arm segment measurements was calculated to be 0.4 Ω.

The trunk was measured to have an R_0_ of 77.9 Ω and an R_∞_ of 41.2 Ω. The SEE for the trunk segment was found to be 0.1 Ω.

The leg was measured to have an R_0_ of 348.6 Ω and an R_∞_ of 231.5 Ω. The SEE of the leg segment was calculated to be 0.3 Ω.

A comparison of the R_0_ and R_∞_ values determined with the three devices is presented in Table 1.

This procedure was repeated with subjects 2–5 for whole-body and segmental measurements. A comparison of the SBISD segmental measurements to the three reference devices, for all five subjects, is presented in Table 2. Each cell shows the percent difference of the SBISD to the reference devices. The largest differences were found when compared with the Seca 514, since the Seca 514 measures from the fingers to the heel, as opposed to wrist to ankle, measuring the bony wrist, hand, and ankle [15].

While the R_0_ and R_∞_ values may differ, it was investigated whether the width of the Cole–Cole curve was similar for the devices. These values are presented in Table 3. The segments as defined in this experiment likely differed from those defined by the Seca, which could contribute to the differences noted.

The commercial analyzers were then compared for their prediction of fat mass, fat-free mass, extracellular fluid, and intracellular fluid. These values are presented in Table 4.

## 4. Discussion

### 4.1. Outcome

The SBISD whole-body measurements were within 5% of both the Multiscan 5000 and SFB7, except for R_∞_ of subject 2, where it differed by 5% from the Multiscan 5000 and 6.8% of the SFB7. For the arm and leg segments, all measurements with the SBISD were within 10% of those performed with the SFB7 and Multiscan 5000. For the trunk, several measurements at R_∞_ differed by more than 10% when compared with either the Multiscan 5000 or the SFB7. In the two cases of a trunk R_∞_ measurement differing by more than 10% from the SFB7, it was within 10% of the Multiscan 5000. For the case where it was more than 10% from the Multiscan 5000, it was within 10% of the SFB7. The R_∞_ values for the trunk was in the 20–40 Ω range, where a 10% error would be a 2–4 Ω difference. Typically, the SBISD had a larger error when determining R_∞_ than R_0_ when compared with the SFB7 or Multiscan 5000.

Trunk measurements led to the most discrepancies between the body composition analyzers. The trunk has a more complex makeup than the arm and leg in terms of the variety and layout of tissues [17]. The Multiscan 5000 and Seca 514 both showed increased error regarding measuring the trunk segments. For the Multiscan 5000, this resulted in less closeness to the circle fit and more spread in some data. The impedance values for the trunk were very close to the lower end of the measuring range of the Multiscan 5000, which was 20–1300 Ω. For the Seca 514, this measuring error was evident due to much larger reactance values in the highest one to three frequencies depending on the subject. The measured trunk values were also very close to the low end of the Seca 514’s impedance measuring range of 10–1000 Ω. Measurement errors and insensitivity to fluid changes in the trunk has been discussed in many articles, so it is an area of interest for segmental measurement capability [11,13,18,19].

### 4.2. How Segmental BIS Overcomes the Disadvantages of Whole-Body BIS

In a review of segmental BIA, it was noted that a drawback of whole-body BIS is the different cross-section sizes of body parts, mainly the limbs versus the trunk [12]. For any given section with length *l* and resistivity R, there will be a linear relationship between FFM and *l*^2^/R. However, for the whole body, this is not the case. Segmental BIA has been used to avoid this variation [7]. There are several conditions where whole-body measurement may not be valid but segmental measurement could be applicable, including for overweight individuals, detecting edemas for early diagnosis, and monitoring hydration in the trunk [7]. Since whole-body BIS is less sensitive to changes in the trunk, it may not be suitable for tracking changes in overweight individuals. A study sought to see whether segmental MF-BIA would be applicable using a standing device with hand grips and foot pads and measuring a variety of BMIs [14]. It was noted that the error compared to DXA increased as %BF increased. For the normal and overweight groups, there was good agreement with a modest error. There was a significant overestimation of %BF for BMIs that were in the obese category. In general, it underestimated %BF for normal BMIs and overestimated it for overweight and obese BMIs. Segmental BIS can allow for tracking changes during recovery from disease, and how each limb is affected. The changes through recovery from dengue fever was monitored in one study [20]. They performed segmental and whole-body measurements on subjects that were either a control group (11 subjects) or a group of dengue fever patients (10 subjects), in the acute and recovery stages. Segmental measurements consisted of the arm, leg, and trunk, and BIS measurements were performed at 50 frequencies and consisted of ECF and ICF resistances. Segmental BIA can provide information about each limb individually, making it suitable for tracking changes in body composition, such as fat and muscle mass, and providing more information about the development of a child. A study aimed to collect data on how fat accumulated in Turkish children with ages between 7 and 18 to provide data for the future diagnosis and tracking of childhood obesity [21]. Measurements were performed on a total of 1371 subjects, measuring arm, leg, and trunk fat, as well as body fat and FFM. Anthropometry was used to determine BMI as a reference. The authors noted that the fat pattern in older children was more central, as opposed to being peripherally located in younger children. They also noted the differences in fat distribution for boys and girls. Whole-body BIA has struggled with the morbidly obese, which was addressed by one study that generated new prediction equations for segmental BIA [22]. The large FM is underestimated by whole-body BIA, while FFM is overestimated. Size and weight restrictions prevent usage of some reference methods, so a portable method is desirable. Segmental and whole-body measurements were taken with the same device at a frequency of 50 kHz, with DXA as the reference method. The FFM provided by the BIA device had a good correlation with that from DXA; however, it did overestimate FFM by 3.9 kg, and this overestimation increased with larger amounts of FFM. For the segments, FM in the trunk was overestimated, while in the limbs, it was underestimated. The effect of the addition of proximal electrodes in segmental BIA was analyzed to see if it could overcome the issue of BIA overestimating %BF in lean elderly and underestimating it in the obese elderly due to their body type differing from that used for prediction equations [23]. The study had 42 subjects and used dilution methods as the reference methods. Different electrode placements were analyzed, including whole body and several segmental models. They found that the addition of proximal electrodes helped to overcome the systematic bias in the %BF predicted by the different electrode placement methods. This was achieved by adding the impedance ratio of proximal limb segments to distal segments.

### 4.3. Comparability with Seca 514

The Seca 514 provided significantly different impedance measurements compared to the other analyzers used, except in the case of some leg segments, particularly the R_∞_ value for the leg measurements. This is because it does not directly measure the segments in the same way. Instead of electrodes at the wrist and ankle for measuring voltage, it has foot pads that measure from the fingers to the heel. This means that it is measuring longer paths through the body, including the high-resistance ankles and wrists [15]. However, when looking at the range of the Cole–Cole curve on the resistance axis, the whole-body curves were generally in agreement, as shown in Table 3. In addition, when comparing the FM, FFM, ECF, and ICF measurements, they were generally within 10% of those found from the other commercial devices. The FM, FFM, ECF, and ICF values for each device are shown in Table 4. This shows that the Seca 514 could be used as a reference device for comparing these values with a tetrapolar BIA device, but not necessarily for comparing impedance measurements directly. In addition to the added wrist and ankle impedance, the differences in limb measurements for the Seca 514 could be explained in part by how it defines and measures the segments. Previous eight-electrode devices used six different current paths: right side of body, left side of body, hand to hand, foot to foot, left hand to right foot, and right hand to left foot. They then used these measurements to determine the impedance for each body segment [24]. For that device, arm and leg segments were defined from the foot to the halfway point in the trunk between the two arms or between the two legs. This means that some of the trunk was included in those measurements. The trunk measurement was determined by subtracting the right arm and right leg from the right side of the body measurement [18]. How the segment measurements were determined by the Seca 514 were not disclosed, but a similar method for trunk impedance must be used.

## 5. Conclusions

A segmental bioelectrical impedance spectroscopy device (SBISD) was designed and a prototype was implemented. The SBISD was then used to measure five subjects, and the results were compared against the results of three commercial devices. It was within 10% for whole-body, arm, and leg measurements, and most trunk measurements when compared with two of the devices, the SFB7 and the Multiscan 5000. The third device used, the Seca 514, could not be compared directly for impedance measurements, but was used to compare the body composition (FM, FFM, ECF, and ICF) measurements of the other commercial devices. Compared to tetrapolar devices like the Multiscan 5000 and the SFB7, the SBISD has the added benefit of measuring three segments simultaneously due to the two additional measuring circuits. It is suggested that future work be done to address the issues in measuring the trunk. This might be addressed by increasing the number of current injection sites to increase the number of current paths so that the different areas with different makeups can be addressed. With the current measuring part of the SBISD validated, further components can be focused on, like signal generation and data acquisition, to create a standalone device.

## Figures and Tables

**Table 1:** Device comparison for subject 1.

		SBISD	MS5000	Seca 514	SFB7
Whole body	R_0_	647.6	662.0	795.0	657.7
	R_∞_	441.2	455.8	575.0	458.6
Arm	R_0_	334.4	335.1	461.6	331.2
	R_∞_	219.2	230.5	330.2	228.3
Trunk	R_0_	75.3	80.1	30.0	77.9
	R_∞_	42.3	36.9	21.3	41.2
Leg	R_0_	333.9	348.3	303.7	348.6
	R_∞_	216.5	229.0	219.9	231.5

**Table 2:** Segmental measurement differences versus SBISD measure.

			% Difference		
			MS5000	Seca 514	SFB7
Subject 1	Whole-Body	R_0_	−2.2	−18.5	−1.5
		R_∞_	−3.2	−23.3	−3.8
	Arm	R_0_	−0.2	−27.6	1.0
		R_∞_	−4.9	−33.6	−4.0
	Trunk	R_0_	−6.0	151.0	−3.3
		R_∞_	14.6	98.6	2.7
	Leg	R_0_	−4.1	9.9	−4.2
		R_∞_	−5.5	−1.5	−6.5
Subject 2	Whole-Body	R_0_	−2.2	−20.9	−1.7
		R_∞_	−5.0	−27.3	−6.8
	Arm	R_0_	−3.0	−33.6	−1.9
		R_∞_	−3.2	−35.4	−5.7
	Trunk	R_0_	−6.6	119.5	−4.4
		R_∞_	9.3	68.7	−19.4
	Leg	R_0_	−3.4	12.3	−1.8
		R_∞_	−4.0	−3.7	−7.9
Subject 3	Whole-Body	R_0_	−1.4	−16.6	−1.5
		R_∞_	−0.7	−20.6	−2.9
	Arm	R_0_	−3.0	−29.1	−1.2
		R_∞_	−1.9	−32.5	−7.9
	Trunk	R_0_	−8.1	187.0	−2.4
		R_∞_	9.0	106.2	−23.5
	Leg	R_0_	−3.4	12.3	−2.3
		R_∞_	−0.8	−1.0	−3.9
Subject 4	Whole-Body	R_0_	−0.8	−15.9	−2.2
		R_∞_	−3.1	−21.8	−4.2
	Arm	R_0_	−0.4	−27.3	−2.0
		R_∞_	−4.7	−30.8	−7.3
	Trunk	R_0_	−4.2	188.2	−3.1
		R_∞_	4.5	116.6	1.4
	Leg	R_0_	−1.9	12.0	−0.8
		R_∞_	−3.4	−1.4	−3.5
Subject 5	Whole-Body	R_0_	−0.6	−16.1	−0.6
		R_∞_	−1.6	−20.9	−1.4
	Arm	R_0_	−1.2	−28.3	−1.3
		R_∞_	−2.3	−31.9	−0.9
	Trunk	R_0_	−6.8	158.8	−3.7
		R_∞_	6.8	105.9	6.1
	Leg	R_0_	−0.8	20.1	−0.4
		R_∞_	−0.1	7.4	−0.3

**Table 3:** Widths of the Cole–Cole Curves.

		R_0_- R_∞_ (Ω)			
		SBISD	MS5000	Seca 514	SFB7
Subject 1	Whole Body	206.4	206.2	220.0	199.1
	Arm	115.2	104.6	131.4	102.9
	Trunk	33.0	43.2	8.7	36.7
	Leg	117.4	119.3	83.8	117.1
Subject 2	Whole Body	220.0	213.4	235.6	202.5
	Arm	93.9	96.6	133.4	88.1
	Trunk	38.9	47.3	12.7	33.6
	Leg	144.0	147.9	98.4	132.9
Subject 3	Whole Body	211.2	217.2	229.4	208.5
	Arm	95.3	100.4	121.6	83.3
	Trunk	47.3	57.7	11.5	38.2
	Leg	132.5	142.9	92.9	131.9
Subject 4	Whole Body	230.5	219.7	227.0	224.7
	Arm	104.3	93.5	126.4	91.9
	Trunk	36.0	41.4	7.4	39.2
	Leg	156.6	155.5	106.3	149.9
Subject 5	Whole Body	148.4	145.7	152.1	146.5
	Arm	79.2	78.3	98.6	80.9
	Trunk	31.4	38.0	9.0	35.6
	Leg	80.2	82.2	47.5	80.7

**Table 4:** Comparison of Body Composition Parameters Determined by the Commercial Analyzers.

		MS5000	Seca 514	SFB7
Subject 1	FM (kg)	13.9	22.0	21.5
	FFM (kg)	66.5	58.4	58.9
	ECF (L)	17.7	17.0	19.2
	ICF (L)	23.8	24.9	23.9
Subject 2	FM (kg)	5.7	9.1	8.2
	FFM (kg)	59.8	56.4	57.3
	ECF (L)	16.5	15.0	17.9
	ICF (L)	25.5	25.5	24.1
Subject 3	FM (kg)	6.8	9.3	10.0
	FFM (kg)	63.8	60.5	60.5
	ECF (L)	17.4	17.0	18.6
	ICF (L)	27.1	26.8	25.7
Subject 4	FM (kg)	12.8	14.3	16.5
	FFM (kg)	46.0	44.5	42.3
	ECF (L)	13.0	13.5	12.9
	ICF (L)	16.9	18.7	18.0
Subject 5	FM (kg)	3.9	6.1	4.6
	FFM (kg)	64.4	62.2	63.7
	ECF (L)	19.4	19.2	21.0
	ICF (L)	25.3	27.1	25.6
